# A Heat Vulnerability Index: Spatial Patterns of Exposure, Sensitivity and Adaptive Capacity for Urbanites of Four Cities of India

**DOI:** 10.3390/ijerph19010283

**Published:** 2021-12-28

**Authors:** Suresh Kumar Rathi, Soham Chakraborty, Saswat Kishore Mishra, Ambarish Dutta, Lipika Nanda

**Affiliations:** 1 Department of Research, MAMTA Health Institute for Mother and Child, New Delhi 110048, India; 2 Indian Institute of Public Health, Public Health Foundation of India, Bhubaneswar 751013, India; soham.c@iiphh.org (S.C.); ambarish.dutta@iiphb.org (A.D.); 3 Centre for Health Care Management, Administrative Staff College of India, Hyderabad 500082, India; saswat@asci.org.in; 4 Department of Multisectoral Planning, Public Health Foundation of India, Gurugram 122002, India; lnanda@iiphb.org

**Keywords:** heat vulnerability index, exposure, sensitivity, adaptive capacity

## Abstract

Extreme heat and heat waves have been established as disasters which can lead to a great loss of life. Several studies over the years, both within and outside of India, have shown how extreme heat events lead to an overall increase in mortality. However, the impact of extreme heat, similar to other disasters, depends upon the vulnerability of the population. This study aims to assess the extreme heat vulnerability of the population of four cities with different characteristics across India. This cross-sectional study included 500 households from each city across the urban localities (both slum and non-slum) of Ongole in Andhra Pradesh, Karimnagar in Telangana, Kolkata in West Bengal and Angul in Odisha. Twenty-one indicators were used to construct a household vulnerability index to understand the vulnerability of the cities. The results have shown that the majority of the households fell under moderate to high vulnerability level across all the cities. Angul and Kolkata were found to be more highly vulnerable as compared to Ongole and Karimnagar. Further analysis also revealed that household vulnerability is more significantly related to adaptive capacity than sensitivity and exposure. Heat Vulnerability Index can help in identifying the vulnerable population and scaling up adaptive practices.

## 1. Introduction

### 1.1. Extreme Heat and Risks

Extreme heat and heat waves are the most under-rated weather phenomena amongst all other extreme weather events (EWEs), i.e., floods, tropical cyclones, heavy rainfalls, cold waves, lightning and so on. This is perhaps the hazardous effects of extreme heat, especially at the onset, are significantly less apparent and discernible. However, extreme heat and heat waves account for more fatalities annually worldwide than all other weather-related hazards combined [1,2]. Further, several studies indicated that the intensity, duration, and frequency of heat waves are likely to increase in a warming climate [1,3]. While extreme heat poses a significant risk to human health in general, threats to the vulnerable populations are particularly worse in an urban setting due to the urban heat island (UHI) effect [2,4,5].

### 1.2. India’s Vulnerability

A study by Carleton et al. (2021) estimates that, in a worst-case scenario, rising temperatures could lead to 221 additional deaths per 100,000 populations each year globally by the year 2100 [6]. The situation appears to be especially grim in the case of the Indian subcontinent. An earlier study by Carleton et al. (2019) stated that around 1.5 million more people may die in India each year due to extreme heat by 2100 [6]. The study further suggested that the average number of extremely hot days is likely to increase to 42.8 degrees Celsius from the current levels of over 35 degrees Celsius. Such a spike in the average summer temperature and the number of extreme hot days significantly increases the risk of mortality. A study by Ray et al. (2021) found that the overall mortality rate for heat waves has increased by a whopping 62.2% over the last 20 years. The study further revealed that as many as 17,362 people were estimated to have been killed due to heat waves during the period of 1971–2019, out of a total of 141,308 deaths ascribed to EWEs, holding a 12.3% share in the total recorded EWEs deaths [7]. During 2012–2018, about 6167 people were killed by heat waves (WHO, 2020). The maximum deaths due to heat waves were registered in the states of Andhra Pradesh, Telangana and Odisha. According to the National Disaster Management Authority—NDMA report (2017), heat waves have caused 25,716 deaths from 1992 to 2016 in various Indian states [8]. A study by Rathi et al. (2021) for Jaipur, India has shown that there can be an excess all-cause mortality by 39% if the temperature exceeds 45 °C [9]. A study from Surat has shown an up to 61% rise in all-cause mortality in some areas of the city during extreme heat situations [10]. Since the year 2000, more people have died from heat waves in India than avalanches, exposure to cold, cyclones, tornadoes, starvation due to natural calamity, earthquakes, epidemics, floods, landslides, torrential rain and forest fires [11]. These mortality figures only refer to what is classified as ‘direct’ deaths due to heat stroke or exertion under direct sunlight. However, the ‘indirect’ or ‘non-exertional’ deaths, which constitute a major share in the total heat-related deaths, are not medically certified as deaths due to heat stroke. Hence, the published data is a gross under-estimation of heat mortality in India. The incidences of heat wave spells have also become more erratic. In the summer of 2019, 73 heat wave spells were recorded against a routine of 17, based on the average during 1986–2016 [7]. Not only the frequency and erraticness, but also the duration of day time hot episodes has increased dramatically over the years across large parts of India [12]. According to the global climate risk index (2021), India is the seventh-worst vulnerable country globally in terms of climate change [13]. The intensity and frequency of periods of extremely hot weather are expected to increase with climate change [14]. Thus, the resultant adverse bearings on human health, lives and livelihoods are expected to be very acute in the Indian context. The poorer segment of the population, constituting about a 28% share [15], is more vulnerable to the worst effects of rising temperatures and likely to bear the brunt more disproportionately.

### 1.3. Heat Vulnerability Indices

Studies have established that vulnerability to heat waves is a combined result of the socio-economic, physiological, climatological, as well as behavioural variables [15,16,17,18]. To understand the concept of vulnerability, it has to be noted that vulnerability is not a static factor which will be common for each household in a particular area. Vulnerability is a very dynamic and fluid state of a person, household or any particular system, which is a combination of multiple other dynamic variables. An increase or decrease in any one of these dynamic variables can lead to a minor or major change in the state of the system, which can either amplify or attenuate vulnerability. These variables can often be classified into exposure, sensitivity and adaptive capacity. While an increase in exposure and sensitivity leads to an increase in vulnerability, but an increase in adaptive capacity leads to a decrease in overall vulnerability [19]. Furthermore, to understand vulnerability to extreme heat, there can be two major approaches. The first approach would include looking at individual factors, like the physiological condition of a person, or climatological conditions and extreme weather phenomena and then predicting their impact on vulnerability. However, for the second approach, a more concrete study will look at every inch of all these phenomena and develop an outcome of these phenomena, which can be a substitute of the quantifiable vulnerability for that particular system (in case of vulnerability to extreme ambient heat, the system is a household). This study is based on a modification of the later approach. Such a unified aggregated index score will not only help policy planners to map household risks to extreme heat but also to highlight relative priority for intervention.

The present study aims to compute a multi-dimensional heat vulnerability index for households in four cities of India, namely, Kolkata in West Bengal, Angul in Odisha, Ongole in Andhra Pradesh and Karimnagar in Telangana State. Any single indicator will only reveal partial information on the vulnerability of households to extreme heat. Use of individual indictors will fail to adequately capture the extent of vulnerability and may be misleading. In order to overcome this problem, a composite index of household vulnerability (HVI) has been constructed following a multidimensional approach. Reports from various domains have suggested that survey-based small-scale analysis with cross-cutting themes should contribute to a better understanding of household-level vulnerability and adaptive capacity, which in turns leads to better mitigation strategies. However, every survey should be very specific to the locale in which it is being conducted. Therefore, this study uses household survey to collect data for construction of HVI [20,21,22,23].

## 2. Materials and Methods

### 2.1. Study Area and Scope

The selection of the cities was based on the recommendations of the Task Force on heat wave by National Disaster Management Authority (NDMA); their geographical location; the Indian Meteorological Department (IMD) criterion of heat wave (temperature ≥45 °C or ≥40 °C) and their representation of Dry (Karimnagar), Hilly (Angul) and Coastal (Kolkata and Ongole) areas.

Each city has a unique characteristic, like the city of Ongole is a coastal urban centre, whereas Karimnagar is completely landlocked. On the other hand, Angul is a major coal mining belt, whereas Kolkata is a metro city and is different from the others in socio-economic capacities [24,25,26,27]. An analytical cross-sectional study has been conducted for vulnerability assessment and the development of heat vulnerability index (HVI) in these four cities. The study conceptualizes household vulnerability as a function of its exposure, sensitivity and adaptive capacity [28]. Exposure, a distinct component of vulnerability, refers to the intensity and spatial distribution of heightened temperature [16], including other factors that elevate heat conditions. Exposure to heat can vary temporally with rising temperature over time or spatially through which some zones of a particular city may be hotter than others. Sensitivity refers to how well a household can cope with increased exposure or the extent to which increased exposure will affect a household physically [15,16]. Adaptive capacity refers to the ability of a household to actively mitigate or adapt to personal exposure [28,29,30], using available skills and resources [31], guaranteeing survival and sustainability [32].

### 2.2. Data and Sampling Design

The study followed a three-stage sampling process with a sample size of 500 households (HHs) from each city. The stratified clustering sampling method was followed for the selection of wards. The first step was listing of the wards of all cities (Angul = 23, Karimnagar = 60, Kolkata = 144 and Ongole = 50) and randomly selecting 25 wards from each city. For Ongole, Karimnagar and Kolkata, 25 wards were selected randomly through IBM SPSS out of the total number of wards for each city. However, for Angul, all the wards were chosen, as there are only 23 municipality wards in the city. In the second step, the slum and non-slum areas from each selected ward were finalized. In the third step, houses were selected from selected slum and non-slum areas by the ground teams of field officers. The first house was selected randomly; then, every 10th house on the right side of the interviewed HHs was included until the target of 20 households from each ward met. Participants from respective households aged 18–60 years were interviewed after taking informed consent.

#### 2.2.1. Data Collection and Management for Household Survey

The vulnerability assessment questionnaire tool for the study was designed using established studies [19,33,34,35]. The variables for this study were selected and divided into several domains to understand the gamut of factors which can lead to an increase in the vulnerability of the population across the four selected cities. The domains in consideration were Socio-economic; Water, Sanitation and Hygiene (WASH); Waste Management; Food and Nutrition; Housing; Locational Characteristics; Community; Risk Perception; Coping Measures; Early Warning; Quality of Life; Co-morbidities; Habits and Livelihood/occupation. Different types of questions existed which helped in increasing the granularity of the data as well as catering to specificities of the study. Types of questions included binary choice (yes or no) questions, multiple choice questions. as well as open-ended questions for selected variables. The questionnaire was extensively reviewed by the experts before field-testing and translated into vernacular (local) languages (Bangla for Kolkata, Oriya for Angul and Telugu for Karimnagar and Ongole). All the selected domains and variables were classified either under exposure, sensitivity or adaptive capacity. The questionnaire was field tested among an eligible non-study population of 30. The individual-level questions were on demographics and health conditions. For individual health condition, the last 15 days history for illnesses including pre-existing and heat-related symptoms and diagnoses were considered.

Household-level questions elicited information on type of house, type of roofing, any load shedding (electricity cut) during the summer, source of water supply for general purposes and drinking and cooling mechanisms in practice. Household-level questions also captured the data on exposure, sensitivity, coping practices and socio-demographic variables like age, sex, income, highest level of education, present major occupation (involved during summer), self-reported pre-existing health conditions, medication history (chronic medication) and any heat-related illnesses and symptoms. The data were collected by trained researchers (field officers) with expertise in community surveys. Before conducting the interview, a participant information sheet was provided and explained to each participant, and written informed consent was obtained from each study participant.

#### 2.2.2. Statistical Analysis

Data were entered in CDC Epi Info (V 7.1.1.6), and all analyses were performed using Microsoft Excel (office 2019), IBM SPSS (V.20) and STATA SE 12 (Stata Corp., College Station, TX, USA). For descriptive purposes, variables were categorized as demographics, exposure variables, sensitivity factors and adaptive behaviours, and city-wise analyses were performed for all variables. Numerical data were expressed as the means and standard deviation (means ± SD).

### 2.3. Approach and Measurement Tools

Heat wave is considered if the maximum temperature of a station reaches at least 40 °C or more for plains and at least 30 °C or more for hilly regions [7]. Vulnerability refers to the diminished capacity of an individual or group to anticipate, cope with, resist and recover from the impact of a natural or man-made hazard, in this case, extreme heat or heat waves [13]. Following the framework of Wilhelmi, [19], this study has defined households’ heat vulnerability as a function of their exposure, sensitivity and adaptive capacity, `as depicted in Equation (1). Each of the three components depends on a range of individual household factors (both qualitative and quantitative) which may influence vulnerability to extreme heat.

Heat Vulnerability = f(Exposure, Sensitivity, Adaptive capacity)(1)

The individual household factors include the presence of people; livelihoods; species or ecosystems; environmental services and resources; infrastructure or economic, social or cultural assets in places that could be adversely affected by an extreme climate event. Exposure refers more to the locational and physical aspects which contribute to an increase in vulnerability [14,15]. Here, the exposure variable is captured through six indicators: (i) tall buildings surrounding the house; (ii) industrial junctions nearby the house; (iii) traffic nearby the house; (iv) roof type; (v) time spent outside and (vi) time spent directly under the sunlight.

Similarly, sensitivity refers to the degree to which a system, asset or species may be affected, either adversely or beneficially, when exposed to climate variability or change or heat- and cold-related hazards [14,15]. Here, the sensitivity variable is captured through eight indicators: (i) age; (ii) annual income; (iii) education level; (iv) presence of hypertension in family members; (v) presence of diabetes in family members; (vi) water shortage; (vii) power-cut and (viii) help from neighbours.

On the other hand, adaptive capacity refers to the ability of systems, institutions, humans and other organisms to adjust to potential damage, to take advantage of opportunities or to respond to consequences of climate or weather extremes [14,15]. The ability of individuals and communities to anticipate, prepare for, reduce the impact of, cope with and recover from the effects of shocks and stresses without compromising their long-term prospects would determine an individual’s or community’s resilience [16]. The adaptive capacity is taken to be influenced by seven indicators: (i) vegetative patches nearby the house; (ii) water bodies nearby house; (iii) wearing summer-appropriate clothes; (iv) reduced time spent outside; (v) drinking more liquid during summer; (vi) use of protective gears such as umbrellas, hats, etc. and (vii) fans/air conditioning system for cooling the home.

Most of the variables have been selected based on empirical evidence from previous studies [17,21,36]. Some variables, like the presence of disabled people in the facility or diseases apart from Diabetes and Hypertension, etc., have also been considered for the study; however, they have been excluded from the index due to the lack of granularity in the survey data.

A detailed description of the variables, their dimensions, measurement and their respective expected impact on the heat vulnerability index is presented in Table 1.

### 2.4. Construction of Multi-Dimensional Vulnerability Index

All the indicators (as depicted in Table 1) have been combined to assess the extent of household heat vulnerability. After identifying the characteristics of heat vulnerability, many studies (cutting across domain areas) have normalized the values to relative positions between 0 and 1 [17,37,38,39] This is done to make the variables unit- and scale-free for comparison purposes. All the dimensions of adaptive capacity have been rescaled to make them directly proportional to heat vulnerability. Hereafter, therefore, the index value for adaptive capacity will be interpreted as a ‘lack of adaptive capacity’.

For the variables that have a positive functional relationship with the respective indicators, normalization has been done using the following formula:

(2) $$d_{i}=\frac{A_{i}-m_{i}}{M_{i}-m_{i}}$$

where *A_i_* = actual value of dimension *i*; *M_i_* = maximum value of dimension *i*; *m_i_* = minimum value of dimension *i*. For the variables that have a negative functional relationship with the respective indicators, normalization has been done using the following formula:

(3) $$di=\frac{M_{i}-A_{i}}{M_{i}-m_{i}}$$

Both these formulae ensure that 0 ≤ *d_i_* ≤1. The higher the value of *d_i_*, higher will be the household’s vulnerability in respect to dimension *i*. When there are *n* dimensions, a household *j* will be represented by a point D_j_ = (d_1_, d_2_,d_3_,…,d_n_) on the *n* dimensional Cartesian space. In the *n*-dimensional space, the point O = O (0,0,0,….0) represents the point of the least vulnerability, whereas the point I = I(1,1,1,…1) represents the highest vulnerability in all dimensions. Following the approach of Wolf et al. (2014) and Aubrecht et al. (2013) [17,40], the multidimensional *HVI* for the *j^th^* household is estimated by the simple average of the component indices for the dimensions. The exact formula is

(4) $$HVI_{j}=\frac{1}{n}\sum_{i=1}^{n}d_{ij}$$

Normalization of indicators and un-weighted quantitative aggregation (additive approach) are common approaches in indicator composition [34]. Alternatively, studies have adopted multivariate statistical techniques, such as principal component analysis and factor or cluster analysis [16,17,18,28].

The (arithmetic) mean HVI for each city has been taken as the cut-off limit to categorize the households into two groups, i.e., high vulnerability and low vulnerability. Households whose HVI values were more than the city’s (average) HVI were grouped under the high vulnerability category. On the contrary, the households whose HVI values were less than the city’s (average) HVI were grouped under the low vulnerability category. Since the distribution of HVI values for all the four cities were found to be normally distributed and without any outliers, the arithmetic mean value has been used as the cut-off criteria.

## 3. Results

### 3.1. Basic Household/Respondent Characteristics

The socio-demographic characteristics of the respondents/households across four surveyed cities are presented in Table 2.

The surveyed population of Ongole has the highest mean age at 42.7 ± 14.8 years, while Angul had the lowest at 37.4 ± 13.0 years. Among the cities, Kolkata has a majority of male respondents, while the other three have a female majority among respondents. The majority of respondents across all the cities were married. The surveyed population of Kolkata had the highest mean household income, followed by Karimnagar and Ongole, while Angul had the lowest. The household expenditure trend corresponded with the household income. Karimnagar had the highest number of households where there was an increase in average expenditure during summer, followed by Ongole, Angul and Kolkata with the least. Kolkata had a majority of graduate or higher qualified respondents, while Ongole had the highest number of respondents who were illiterate (Table 2).

### 3.2. Household Exposure to Extreme Heat

Distribution of the sampled households by exposure to extreme heat across four cities is presented in Table 3.

Kolkata had the highest number of houses surrounded by tall buildings on either three or four sides (>52%), leading to a blockage in air-flow. Kolkata is followed by Angul, Ongole and Karimnagar in terms of households surrounded by tall buildings on 3 or 4 sides. Angul had the highest number of households with a roof made of asbestos or tin (~40%), followed by Ongole, Karimnagar and Kolkata. Kolkata also had the highest mean hours of respondents being exposed to direct sunlight (Table 3).

### 3.3. Household Sensitivity to Extreme Heat

The distribution of the sampled households by sensitivity to extreme heat across four cities is presented in Table 4.

All the cities had a relatively similar number of respondents and family members who were suffering from Hypertension and Diabetes. Many households (~48%) faced a water shortage during the summer months in Ongole, followed by Angul, Karimnagar and Kolkata. In terms of power cuts during summer, Angul had the highest number of households reporting the same, followed by Ongole, Karimnagar and Kolkata. Angul also had the highest number of respondents who mentioned getting no help from neighbours during an emergency, followed by Karimnagar, Ongole and Kolkata (Table 4).

### 3.4. Household Adaptive Capacity to Extreme Heat

The distribution of the sampled households by adaptive capacity to extreme heat across four cities is presented in Table 5.

Kolkata had highest number of households either near a water body or vegetative patches, followed by Ongole, Angul and Karimnagar. More than 36% of the population in all the cities wear summer appropriate cloths during extreme summer days to protect themselves from the heat. A majority of respondents in Ongole, Karimnagar and Angul (~65–80%) reduce the time spent outside during summer months, while only 4.4% of respondents do the same in Kolkata. All the four cities rely mostly on drinking lots of fluid, using umbrella/hats and using fans/Air Conditioners /coolers to protect them from extreme summer (Table 5).

### 3.5. Multi-Dimensional Household Heat Vulnerability Index

Using the data on all three dimensions (exposure, sensitivity and adaptive capacity) for the sampled households, the HVI scores were computed for all the four cities (Kolkata, Angul, Ongole and Karimnagar). The distribution of the sampled households by HVI values across the four cities is presented in Table 6.

#### 3.5.1. Kolkata

**Figure 1:**
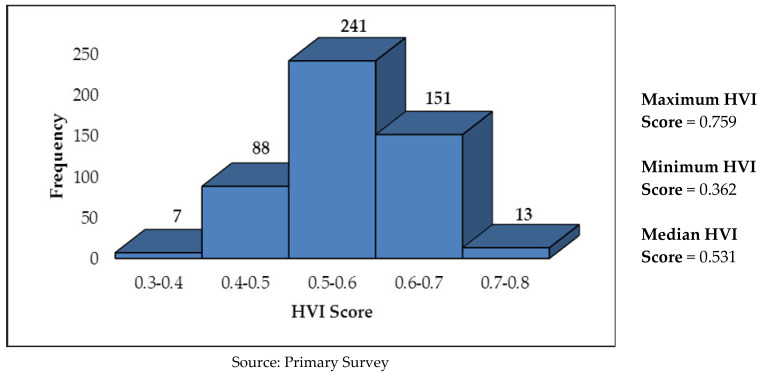
Pictorial Distribution of No. of Households by HVI Scores in Kolkata.

In Kolkata, overall, 67.2% of the sample households have a high vulnerability to extreme heat, whereas the rest (32.8%) have a low vulnerability. Maximum vulnerability was seen in the sensitivity parameter, wherein as many as 77.2% of the households are found to be highly vulnerable, followed by exposure (73.0%) and lack of adaptive capacity (38.6%). The pictorial distribution of households in Kolkata by HVI scores (range) is given in Figure 1. While the median HVI score in Kolkata is 0.531, as high as 164 households (32.8%) have HVI score of more than 0.6.

#### 3.5.2. Angul

**Figure 2:**
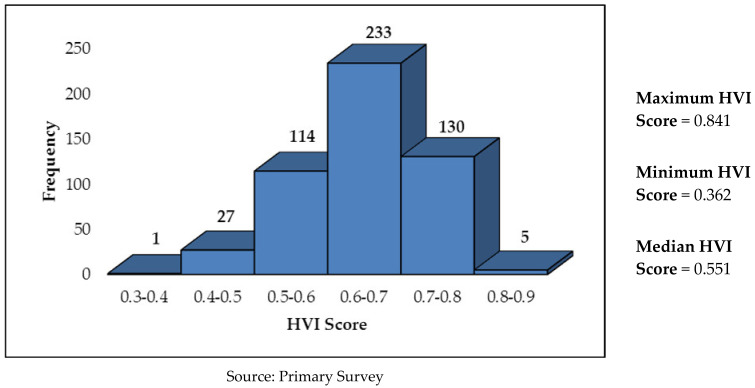
Pictorial Distribution of No. of Households by HVI Scores in Angul.

In Angul, overall, 73.5% of the sample households have a high vulnerability to extreme heat, whereas the rest (26.5%) have a low vulnerability. Maximum vulnerability is seen in the sensitivity parameter, wherein as many as 93.3% of the households were found to be highly vulnerable, followed by exposure (51.0%) and lack of adaptive capacity (34.7%). The pictorial distribution of households in Angul by HVI scores (range) is given in Figure 2. While the median HVI score in Angul is 0.551, as many as 368 households (72.2%) have HVI score of more than 0.6.

#### 3.5.3. Ongole

**Figure 3:**
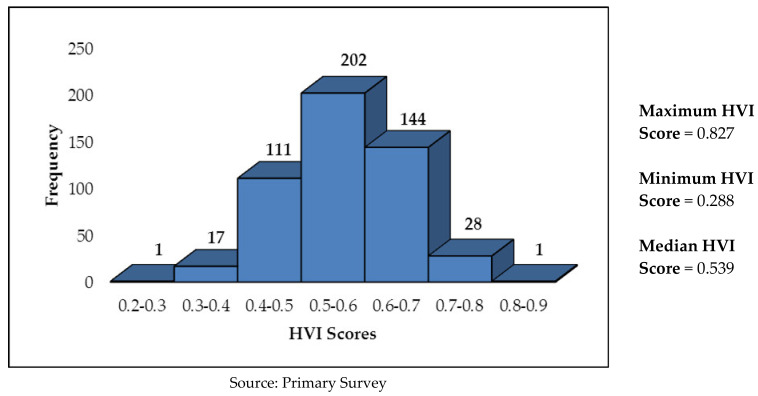
Pictorial Distribution of No. of Households by HVI Scores in Ongole.

In Ongole, overall, 65.7% of the sample households have a high vulnerability to extreme heat, whereas the rest (34.3%) have a low vulnerability. Maximum vulnerability was observed in sensitivity parameter, wherein as many as 68.3% of the households were found to be highly vulnerable, followed by lack of adaptive capacity (57.3%) and exposure (51.6%). The pictorial distribution of households in Ongole by HVI scores (range) is given in Figure 3. While the median HVI score in Ongole is 0.539, 173 households (34.3%) have HVI score of more than 0.6.

#### 3.5.4. Karimnagar

**Figure 4:**
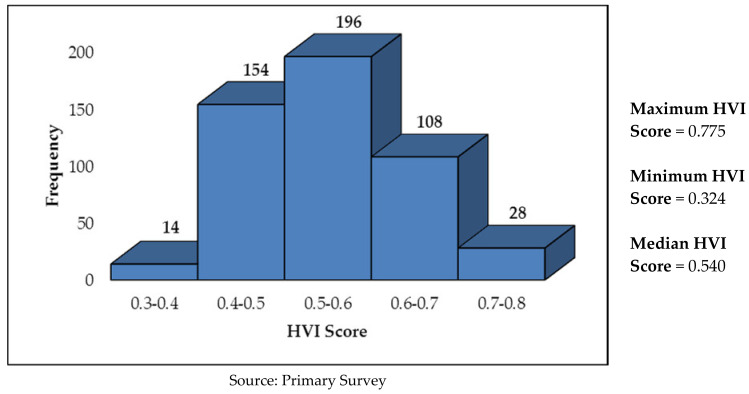
Pictorial Distribution of No. of Households by HVI Scores in Karimnagar.

In Karimnagar, overall, 66.4% of the sample households have a high vulnerability to extreme heat, whereas the rest (33.6%) have a low vulnerability. Maximum vulnerability was observed in sensitivity parameter, wherein as many as 72.2% of the households were found to be highly vulnerable, followed by exposure (52.0%) and lack of adaptive capacity (49.8%). The pictorial distribution of households in Karimnagar by HVI scores (range) is given in Figure 4. While the median HVI score in Karimnagar is 0.540, 136 households (27.2%) have HVI score of more than 0.6.

Table 7 shows the pair-wise correlation between the different components of HVI. The results show that HVI is significantly correlated with a lack of adaptive capacity. Therefore, an increase in adaptive capacity will lead to a drop in HVI.

## 4. Discussion

The study attempted to aggregate multiple well-known heat-related indicators to construct a multidimensional heat vulnerability index (HVI) for urban households across four cities of India, i.e., Angul, Kolkata, Ongole and Karimnagar. While Angul and Kolkata are located in the eastern belt of India, Ongole and Karimnagar are situated in the southern part of the country. This study applied an inductive approach for the development of a heat vulnerability index for four Indian Cities. To our knowledge, this is the first ever study reporting the development of a multidimensional heat vulnerability Index for Indian Cities. Conceptualizing heat vulnerability as a function of exposure, sensitivity and adaptive capacity [28], the study computed the multidimensional HVI for each household to assess the magnitude of their vulnerability to extreme heat and heat waves.

The developed HVI indicated that majority of the urban households in Indian cities have a high vulnerability to extreme heat. At the regional level, the urban households in the southern part of India have a relatively lower overall heat vulnerability as compared to those in the eastern belt of the country. This is because both exposure and sensitivity are comparatively lower among the urban households of the southern cities. On the contrary, the adaptive capacity of urban households to counter extreme heat is lower in southern cities as compared to those in eastern cities. This multidimensional HVI approach enables one to capture information on several dimensions in a ‘single’ metric. The major components of the index can help in recognizing the underlying characteristics for heat vulnerability. It can also serve to monitor the progress of policy initiatives aimed at reducing vulnerability of households in the sampled cities over time. However, there was also considerable variation in the adaptive capacity of households among the cities. The results of the Pearson correlation matrix established a positive (statistically) significant linkage between overall heat vulnerability and lack of adaptive capacity. This result further corroborates the findings of Laranjeira et al. (2021), Hayden et al. (2017), Hess et al. (2012) and OECD (2009) [20,41,42,43]. It implies that heat vulnerability can be controlled in the presence of more exposure and sensitivity if an adequate adaptive capacity is built. The governments must, therefore, take up short-term measures for enhancing the adaptive capacity of the vulnerable households in the surveyed cities. Long-term strategies can be designed towards reducing the exposure and sensitivity of the vulnerable groups. In terms of heat mitigation, several suggestions have been proposed by scientists worldwide, which also include large-scale solutions like the Superblock Model [44].

The pairwise correlation coefficient between overall vulnerability to extreme heat and lack of adaptive capacity was found to be significantly strong and positive for the households in Angul (0.77), Kolkata (0.75) and Karimnagar (0.78). It was moderately strong and positive in case of Ongole (0.67). Study by Inostroza, L, et.al., (2016) have also shown a strong correlation of adaptive capacity with HVI [45].

Existing studies [29,46,47,48] define the relationship between adaptive capacity and vulnerability in three ways. First, vulnerability and adaptive capacity are not mutually exclusive. Second, vulnerability is the consequence of a lack of adaptive capacity, among several other factors. Third, both are inversely proportional, so that high capacity entails low vulnerability and vice-versa. In case of this study, it was found that vulnerability is (directly) inversely proportionate to (lack of) adaptive capacity. This relationship was also found to be highly significant with the pair-wise correlation. The pair-wise correlation coefficient values signify that even if exposure and sensitivity is high, the overall vulnerability to extreme heat can be contained by improving adaptive capacity.

**Limitations:** a few points merit attention:

- The indicators selected for this analysis might not be universally applicable as they are highly locale-specific; a similar sort of index with universal indicators might not be able to capture the gamut of dimensions which add to vulnerability.
- The primary data collection work involving the survey of 2000 households during the COVID-19 pandemic posed significant challenges. The situation compelled the authors to make multiple modifications in the sampling strategy to obtain the best quality of data.
- Being a cross-sectional study, some inherited biases cannot be ignored, such as re-porting and recall bias and interviewer bias.
- As we asked respondents about health conditions of other members, illness history and medication practices, some extent of under-reporting cannot be ignored, which may have an impact on the findings.

## 5. Conclusions

Angul has the maximum share of households that are vulnerable to extreme heat, followed by Kolkata, Karimnagar and Ongole. While sensitivity is higher in Angul, exposure is more in Kolkata as compared to the other two cities. Ongole has the maximum share of households with a lack of adaptive capacity, followed by Karimnagar and Kolkata. On the other hand, Angul is home to the least share of households with a lack of adaptive capacity. The computed HVI for each household can be used to reveal spatial hotspots of the perceived heat stress within each city. It could also assist the heat warning systems and local government in targeting high-risk areas and protecting people’ s health more effectively on extreme heat days. This multidimensional HVI could be utilized by local government city officials, urban planners, the disaster management authority and the public health department to aid in the mitigation of extreme heat events. The study results further highlight the need to understand adaptive capacities at the household level and identify what limits households’ capacity to adapt to extreme heat and heatwaves.

The HVI, by itself, only serves the purpose of identifying who the most vulnerable are? However, for a deeper understanding, the HVI can be used with more advanced statistical models to identify the factors which accentuate or minify overall heat vulnerability.

The socio-economic, health and ecological factors which influence both the incidence and extent of HVI leaves a potential area for future research. Furthermore, how HVI and adaptive capacity (sub-index value) vary across socio-economic, demographic and occupational groups (besides the role of technology and institutions) require further empirical scrutiny.

Similar exercises with locale-specific variables should be conducted by each city, especially for the cities which are more prone to heat-wave-like situations. Assessment of temperature thresholds beyond which there is a rise in all-cause mortality should be conducted by every city, and a vulnerability analysis should be planned accordingly. The vulnerability of household data can also be used to prepare vulnerability maps [33]. New studies should also consider the period of COVID-19 to analyse the impact of the pandemic on urban heat vulnerability and develop plans accordingly, as also recommended in several studies [49,50].

## Figures and Tables

**Table 1:** Description of variables and expected impact on vulnerability.

Dimension	Indicator	Measurement	Expected Impact on Vulnerability
Exposure	Tall buildings	Tall buildings are defined by the total number of sides of the house that is surrounded by tall buildings	Positive
	Industrial junctions	Industrial junction is defined as a dummy variable. It takes a value 1 if there are any factories or major industrial areas nearby the house and 0 otherwise	Positive
	Traffic Junctions	Traffic junction is defined as a dummy variable. It takes a value 1 if there is any highway or heavy traffic junction nearby the house and 0 otherwise	Positive
	Roof type	Roof type is categorized into five groups as stated below: 1 = concrete; 2 = Asbestos; 3 = Clay tiles; 4 = Tin-sheet; 5 = Straw	Positive
	Time spent outside	It is defined as the number of hours spent outside in a day on average by the household	Positive
	Time spent under direct sunlight	It is defined as the number of hours spent directly under sunlight in a day on average by the household	Positive
Sensitivity	Age	It is measured by the mean age of the household (in number of years)	Positive
	Annual income	It is measured by the annual average income of the household (in INR)	Negative
	Education level	Education level is categorized into six groups as stated below: 0 = Illiterate; 1 = Primary; 2 = Middle; 3 = High School; 4 = Intermediate; 5 = Graduation; 6 = Other professional course	Negative
	Hypertension	It is measured by the number of household members who have hypertension	Positive
	Diabetes	It is measured by the number of household members who have diabetes	Positive
	Water shortage	Water shortage is defined as a dummy variable. It takes a value 1 if the household faces water shortage and 0 otherwise	Positive
	Power-cut	Power-cut is defined as a dummy variable. It takes a value 1 if the household faces power-cuts in the summers and 0 otherwise	Positive
	Help from neighbours	Help is defined as a dummy variable. It takes a value 1 if the household receives any form of help from the neighbours and 0 otherwise	Positive
Adaptivity	Vegetative patches	Vegetative patches are defined as a dummy variable. It takes a value 1 if the household has any vegetative patches, like parks, fields, etc., nearby their house and 0 otherwise	Negative
	Water bodies	Water bodies are defined as a dummy variable. It takes a value 1 if the household has any medium to large water bodies like ponds, lakes, rivers, etc., nearby their house and 0 otherwise	Negative
	Summer clothes	Summer clothes are defined as a dummy variable. It takes a value 1 if the household members wear summer-appropriate clothes and 0 otherwise	Negative
	Reduced time	Reduced time is defined as a dummy variable. It takes a value 1 if the household members have reduced time spent outside during summer and 0 otherwise	Negative
	Drinking more liquid	Drinking more liquid is defined as a dummy variable. It takes a value 1 if the household members have increased the intake of liquids in the summer months to deal with heat and 0 otherwise	Negative
	Protective gears	Use of protective gears is defined as a dummy variable. It takes a value 1 if the household members use umbrellas/hats/head-covers to prevent direct sunlight and 0 otherwise	Negative
	Cooling home	Cool home is defined as a dummy variable. It takes a value 1 if the household uses fans or Air Conditioners as a mode to keep their home cooler and 0 otherwise	Negative

**Table 2:** Socio-demographic characteristics for the population of four Indian Cities.

Variable	Ongole	Karimnagar	Kolkata	Angul
No. of Households	504	500	500	510
Age	42.7 ± 14.8	38.6 ± 15.0	39.6 ± 13.1	37.4 ± 13.0
Years in the city	32.5 ± 17.7	33.4 ± 16.8	6.5 ± 16.6	24.5 ± 14.2
Households with a change of income in extreme summer (%)	116 (23.0)	132 (26.4)	66 (13.2)	222 (43.5)
Change in monthly expenditure in summer				
Increased	437 (86.7)	440 (88.0)	95 (19.0)	328 (64.3)
Decreased	5 (1.0)	5 (1.0)	25 (5.0)	13 (2.5)
No Change	62 (12.3)	55 (11.0)	380 (76.0)	169 (32.1)
Gender				
Male	171 (33.9)	231 (46.2)	321 (64.2)	173 (33.9)
Female	333 (66.1)	266 (53.2)	174 (34.8)	334 (65.5)
Transgender	0 (0)	3 (0.6)	5 (1.0)	3 (0.6)
Religion				
Hinduism	259 (51.4)	445 (89.0)	469 (93.8)	502 (98.4)
Christianity	60 (11.9)	26 (5.2)	2 (0.4)	1 (0.2)
Islam	179 (35.5)	28 (5.6)	21 (4.2)	5 (1.0)
Others	6 (1.2)	1 (0.2)	8 (1.6)	2 (0.4)
Households with pregnant women	6 (1.2)	6 (1.2)	19 (3.8)	9 (1.8)
Marital status				
Single	26 (5.2)	80 (16.0)	38 (7.6)	4 (0.8)
Unmarried	26 (5.2)	47 (9.4)	96 (19.2)	68 (13.3)
Married	375 (74.4)	338 (67.6)	332 (66.4)	395 (77.3)
Separated	1 (0.2)	4 (0.8)	1 (0.2)	2 (0.4)
Divorced	4 (0.8)	6 (1.2)	8 (1.6)	7 (1.4)
Widowed	59 (11.7)	21 (4.2)	24 (4.8)	35 (6.9)
No response	13 (2.6)	4 (0.8)	1 (0.2)	0 (0)
Education Level				
Illiterate	196 (38.9)	104 (20.8)	8 (1.6)	124 (24.5)
Primary School Certificate	34 (6.7)	17 (3.4)	20 (4.0)	73 (14.3)
Middle School Certificate	63 (12.5)	43 (8.6)	51 (10.2)	84 (16.5)
High School Certificate	91 (18.1)	70 (14.0)	110 (22.0)	129 (25.3)
Intermediate or post HS Diploma	54 (10.7)	81 (16.2)	52 (10.4)	44 (8.6)
Graduate/Post-graduate/Professional/Honours	64 (12.7)	180 (36.0)	252 (50.8)	51 (10.0)
No Response	2 (0.4)	5 (1.0)	5 (1.0)	4 (0.8)

**Table 3:** Descriptive Statistics: Exposure.

Variable	Ongole	Karimnagar	Kolkata	Angul
Households surrounded by tall buildings				
One Side	47 (9.3)	135 (27.0)	46 (9.2)	40 (7.8)
Two Sides	39 (7.7)	83 (16.6)	173 (34.6)	117 (28.9)
Three Sides	40 (7.9)	14 (2.8)	143 (28.6)	102 (20.0)
Four Sides	9 (1.8)	7 (1.4)	120 (24.0)	48 (9.4)
None	369 (73.2)	261 (52.2)	18 (3.6)	198 (38.8)
Presence of locational characteristics				
Industrial areas	91 (18.1)	27 (5.4)	27 (5.4)	58 (11.4)
Traffic junctions	107 (21.2)	68 (13.6)	220 (44.0)	46 (9.0)
Type of roof				
Concrete	262 (52.0)	344 (68.8)	358 (71.6)	243 (47.6)
Asbestos	179 (35.5)	50 (10.0)	70 (14.0)	193 (37.8)
Clay tiles	45 (8.9)	42 (8.4)	39 (7.8)	30 (5.9)
Tin sheds	5 (1.0)	46 (9.2)	21 (4.2)	10 (2.0)
Straw	7 (1.4)	9 (1.8)	0 (0.0)	31 (6.1)
Others	6 (1.2)	8 (1.6)	12 (2.4)	3 (0.6)
Hours spent outside	3.54 ± 3.73	3.76 ± 3.95	6.28 ± 4.15	3.39 ± 2.76
Hours spent outside in direct sunlight	1.25 ± 1.70	1.74 ± 2.38	3.42 ± 5.4	1.99 ± 2.43

**Table 4:** Descriptive Statistics: Sensitivity.

Variable	Ongole	Karimnagar	Kolkata	Angul
Co-morbidities				
Hypertension	140 (8.2)	150 (9.3)	100 (6.5)	143 (8.0)
Diabetes	129 (7.5)	100 (6.2)	135 (9.6)	76 (4.2)
Water shortage				
In normal days	80 (15.9)	36 (7.2)	28 (5.6)	64 (12.5)
In extreme summer days	240 (47.6)	107 (21.4)	50 (10.0)	138 (27.1)
Power cut				
In normal days				
Yes	22 (4.4)	40 (8.0)	14 (2.8)	132 (25.9)
No	482 (95.6)	451 (90.2)	485 (99.0)	378 (74.1)
No response	0 (0)	9 (1.8)	1 (0.2)	0 (0)
In Summer days				
Yes	118 (23.4)	82 (16.4)	18 (3.6)	487 (95.5)
No	386 (76.5)	408 (81.6)	479 (95.8)	22 (4.3)
No response	0 (0)	10 (2.0)	3 (0.2)	1 (0.2)
Help from extended family				
Yes	419 (83.1)	406 (81.2)	434 (86.8)	415 (81.4)
No	69 (13.7)	73 (14.6)	29 (5.8)	93 (18.2)
May be	16 (3.2)	21 (4.2)	37 (7.4)	2 (0.4)

**Table 5:** Descriptive Statistics—Adaptive Capacity.

Variable	Ongole	Karimnagar	Kolkata	Angul
Presence of locational characters				
Vegetative patches	238 (47.2)	37 (7.4)	370 (74.0)	26 (5.1)
Water bodies	167 (33.1)	58 (11.6)	237 (47.5)	103 (20.2)
Wearing different type of clothing during summer than during regular time				
Yes	189 (37.5)	217 (43.4)	188 (37.6)	186 (36.5)
No	315 (62.5)	283 (56.6)	312 (62.4)	324 (63.5)
Time spent outside during summer				
Increased	4 (0.8)	4 (0.8)	12 (2.4)	2 (0.4)
Decreased	324 (64.3)	346 (69.2)	22 (4.4)	407 (79.8)
No Change	176 (34.9)	150 (30.0)	466 (93.2)	101 (19.8)
Coping Measures				
More Liquid	280 (55.5)	432 (86.4)	318 (64.6)	399 (78.2)
Umbrella/hat	311 (61.7)	354 (70.8)	324 (64.8)	327 (64.1)
Fan/AC	442 (87.7)	455 (91.0)	310 (62.0)	341 (66.9)

**Table 6:** Household Vulnerability for four Indian Cities (Percentage Share).

Vulnerability	Kolkata			Angul			Ongole			Karimnagar		
	High	Low	Total	High	Low	Total	High	Low	Total	High	Low	Total
Overall HVI	336 (67.2)	164 (32.8)	500 (100)	375 (73.5)	135 (26.5)	510 (100)	331 (65.7)	173 (34.3)	504 (100)	332 (66.4)	168 (33.6)	500 (100)
Exposure	365 (73.0)	135 (27.0)	500 (100)	260 (51.0)	250 (49.0)	510 (100)	260 (51.6)	244 (48.4)	504 (100)	260 (52.0)	240 (48.0)	500 (100)
Sensitivity	386 (77.2)	114 (22.8)	500 (100)	476 (93.3)	34 (06.7)	510 (100)	344 (68.3)	160 (31.7)	504 (100)	361 (72.2)	139 (27.8)	500 (100)
Lack of Adaptive Capacity	193 (38.6)	307 (61.4)	500 (100)	173 (34.7)	333 (65.3)	510 (100)	289 (57.3)	215 (42.7)	504 (100)	249 (49.8)	251 (50.0)	500 (100)

Source: Authors’ computations based on data from primary survey.

**Table 7:** Pair-wise Correlation between different Components of HVI.

		Exposure	Sensitivity	Lack of Adaptive Capacity	HVI
Kolkata	Exposure	1			
	Sensitivity	0	1		
	Lack of Adaptive Capacity	−0.12 ***	0.01	1	
	HVI	0.44 ***	0.38 ***	0.75 ***	1
Angul	Exposure	1			
	Sensitivity	0	1		
	Lack of Adaptive Capacity	−0.05	−0.13 ***	1	
	HVI	0.43 ***	0.32 ***	0.77 ***	1
Ongole	Exposure	1			
	Sensitivity	−0.05	1		
	Lack of Adaptive Capacity	−0.06	0	1	
	HVI	0.44 ***	0.54 ***	0.67 ***	1
Karimnagar	Exposure	1			
	Sensitivity	−0.04	1		
	Lack of Adaptive Capacity	0.1	0.01	1	
	HVI	0.51 ***	0.44 ***	0.78 ***	1

Note: *** Significant at 1% level of significance.

## Data Availability

Survey data are available from authors. We need approval from the Government of India (National Disaster Management Authority of India) for sharing the data.
